# Long-term incidence and outcomes of obesity-related peripheral vascular disease after bariatric surgery

**DOI:** 10.1007/s00423-020-02066-9

**Published:** 2021-01-12

**Authors:** Osama Moussa, Maddalena Ardissino, Silvia Muttoni, Ara Faraj, Alice Tang, Omar Khan, Peter Collins, Usman Jaffer, Sanjay Purkayastha

**Affiliations:** 1 Department of Surgery and Cancer, Imperial College London, Praed Street, London, W2 1NY UK; 2 Department of Medicine, Imperial College London, London, SW7 2AZ UK; 3 Department of Upper GI and Bariatric Surgery, St George’s University of London, London, SW17 0RE UK; 4 Academic Unit, Imperial College London School of Medicine, Exhibition Road, London, SW7 2AZ UK; 5 Royal Brompton Hospital and National Heart and Lung Institute, Imperial College London, London, SW3 6NP UK; 6 Department of Vascular Surgery, St Mary’s Hospital, Imperial College NHS Trust, Praed Street, London, W2 1NY UK

**Keywords:** Peripheral vascular disease, Venous insufficiency, Arterial disease, Ischaemia, Bariatric surgery, Obesity

## Abstract

**Background and aims:**

Patients with obesity are at high risk of suffering from arterial and venous peripheral vascular disease (PVD). Bariatric surgery is an effective strategy to achieve weight reduction for patients with obesity. The long-term impact of bariatric surgery on obesity-related morbidity is subject to increasing research interest. This study aimed to ascertain the impact of bariatric surgery on the long-term occurrence of PVD in patients with obesity.

**Methods:**

The study population was extracted from the Clinical Practice Research Datalink, a nation-wide database containing primary and secondary care records of consenting patients. The intervention cohort was 2959 patients who had undergone bariatric surgery during follow-up; their controls were 2959 propensity-score-matched counterparts. The primary endpoint was development of any PVD: arterial or venous. Secondary endpoints were incident peripheral arterial disease alone, incident peripheral venous disease alone.

**Results:**

Three hundred forty-six patients suffered a primary endpoint during follow-up. Bariatric surgery did not improve peripheral vascular disease rates as a whole, but it was associated with significantly lower event rates of arterial disease (HR = 0.560, 95%CI 0.327–0.959, *p* = 0.035) but higher event rates of venous disease (HR = 1.685, 95%CI 1.256–2.262, *p* < 0.001).

**Conclusions:**

Bariatric surgery was associated with significantly reduced long-term occurrence of arterial disease but increased occurrence of venous disease in patients with obesity.

**Supplementary Information:**

The online version contains supplementary material available at 10.1007/s00423-020-02066-9.

## Introduction

Since the beginning of its clinical development and application in the 1950s, bariatric surgery has steadily grown in popularity as a treatment option for patients with obesity [1]. In recent years, its use experienced a further surge that can be broadly related to three main reasons. Firstly, the development of endoscopic and minimally invasive techniques has allowed faster and safer intervention with lower risk of complications. Secondly, an ever-increasing volume of data has validated it as an extremely effective method of achieving weight reduction and, perhaps most importantly, of controlling some of its most severe and life-threating comorbidities such as hypertension, dyslipidaemia, and diabetes [2–4]. Thirdly, the worldwide rates of obesity are steeply rising, creating a truly global epidemic, and causing unprecedented rates of obesity-related morbidity and mortality [5].

The detrimental health effects of obesity are numerous and have been previously extensively explored. Vascular complications are among the most common, including peripheral arterial and venous disease. The correlation between obesity and arterial vascular morbidity may be mediated by the increased inflammation [6] and increased incidence of risk factors for atherosclerosis which are associated with obesity: type 2 diabetes, hypertension, and dyslipidaemia. Bariatric surgery is known to improve the control of, and even induce resolution of metabolic and atherosclerotic vascular risk factors, [3, 7–9] suggesting a potential role in the reduction of long-term risk of arterial disease. With regards to venous disease, the potential haemodynamic effects of decreased intra-abdominal pressure [10, 11], secondary to weight loss from bariatric surgery, may feasibly improve venous return and therefore decrease the risk of lower limb venous insufficiency and thrombosis. For these reasons, it is reasonable to hypothesize that beyond its effect on weight, bariatric surgery may be beneficial in reducing the risk of peripheral arterial and lower limb venous morbidity in patients with obesity.

Despite the established correlation between obesity and both peripheral arterial and lower limb venous disease, this clinical question has not previously been addressed. The aim of this nation-wide cohort study is to ascertain the impact of bariatric surgery on the long-term incidence of peripheral arterial and lower limb venous morbidity in patients with obesity.

## Methods

### Study database

The data analysed in this study was acquired from the Clinical Practice Research Datalink (CPRD) database, a UK-based governmental research database funded by the National Institute for Health Research (NIHR) and the Department of Health (DoH). Informed consent was obtained from all individual participants included in the study. The data contained within CPRD is anonymised and continuously updated, and has been demonstrated to be an accurate representation of the British population in terms of ethnicity and demographics [12, 13]. The design, implementation, and maintenance of this database have been previously described [13, 14].

### Ethical approval

Scientific approval for this study was granted by The Regulatory Agency’s Independent Scientific Advisory Committee (ISAC approval registration number: 16_140R2). Ethical approval was granted by the Health Research Authority IRAS. Project ID: 203143.

### Patient population

**Fig. 1:**
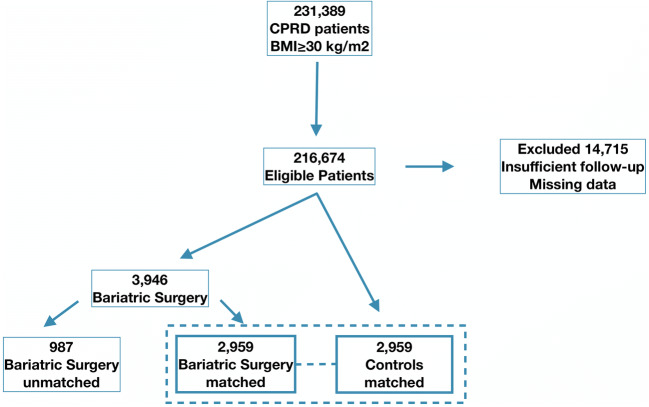
Patient selection flowchart

A retrospective, nested cohort study was designed. Inclusion criteria was limited to the 231,389 patients that had a recorded body mass index (BMI) of ≥ 30 kg/m^2^ in their healthcare record before cohort inclusion. A total of 14,715 patients were excluded from the analysis due to insufficient follow-up time to establish long-term outcomes (< 6 months), missing information on BMI, gender, or age. Patients who had a pre-existing diagnosis of peripheral arterial or lower limb venous disease before enrollment in either study arm were also excluded. This resulted in a total of 216,674 patients considered for analysis. Bariatric surgery patients were then matched on s 1:1 basis with equal numbers of control on the basis of a propensity score that included all baseline demographic (age, gender, BMI) and morbidity (hyperlipidaemia, hypercholesterolaemia, diabetes, atrial fibrillation, smoking) variables. A total of 2959 bariatric surgery patients were successfully matched with a caliper of 0.05, and 987 could not be matched due to missing information (the patient selection process is outlined in Fig. 1.

The study variables were defined as follows. Gender refers to the gender of the participants as coded in the demographic information in the database. Age refers to the age at the time of index date (the year of bariatric surgery in the intervention cohort, and the year of entry to the database for the control cohort). Baseline BMI reading refers to the BMI reading closest in time to the date of indexing.

Demographic details of all included patients were noted, specifically the baseline BMI at the time of first diagnosis of obesity, gender, age, the presence of several morbidity, and treatment factors at the time of index date (hypertension, hyperlipidaemia, diabetes mellitus, atrial fibrillation, smoking, alcohol) as well as the use of a number of medications (warfarin, direct oral anticoagulants, aspirin, beta blockers, angiotensin-converting enzyme inhibitors).

### Endpoint definition

The primary endpoint of this study was a composite of occurrence of any peripheral arterial disease or peripheral venous disease.

Secondary endpoints included the individual evaluation of occurrence of the following:

- Peripheral arterial disease alone
- Peripheral venous disease alone

Peripheral arterial disease was defined as the presence of any of the following diagnoses: claudication, arterial insufficiency, acute or chronic limb ischaemia, arterial ulcers or a recorded ABPI < 0.8.

Peripheral venous disease was defined by the presence of a diagnosis of venous insufficiency, venous ulcers, venous eczema, or varicose veins.

Repeat events were not included in the analysis for either primary or secondary endpoints. Both binary and time-to-event data were extracted for all outcomes. Event rates are expressed in both overall percentages, and in rates per 10,000 person-years (/10000 py) in view of the different lengths of follow-up across cohorts.

### Statistical analysis

Statistical Package for Social Sciences, version 25 was used for all data extraction and preparation (SPSS Inc., New York, NY, USA) [15]. The data for the study was analysed using RStudio (Version 1.2.5033 © 2009-2019 RStudio, Inc.).

Baseline demographic, clinical, and treatment factors were compared between study arms using the Pearson’s chi-squared test for categorical variables. Hazard data was analysed for both primary and secondary endpoints using a Cox proportional hazards model that was adjusted for multiple covariates. All factors found to be imbalanced across cohorts were adjusted for in the models. Interaction analysis was performed using a Cox proportional hazards model with interaction terms. Two sensitivity analyses were performed. Firstly, one without any covariate adjustment. Secondly, one including only patients who underwent gastric band or bypass as their bariatric surgery.

All *p* values reported are 2-sided; and statistical significance was considered when *p* < 0.05.

## Results

### Baseline characteristics

Five thousand nine hundred and eighteen (5,918) were included in the study. Of these, 2,959 had undergone bariatric surgery and 2,959 propensity-score-matched controls had not. The mean length of total follow-up for all patients was 91.6 months (52.7 ± 39.8 bariatric surgery cohort, 130.3 ± 85.6 controls) equating to 7.6 years. The propensity score density plots before and after matching are depicted in Supplementary Figure 1.

The mean age at first recorded diagnosis of obesity was 44.7 (44.6 ± 10.6 bariatric surgery cohort, 44.8 ± 13.1 controls). Patients who underwent bariatric surgery achieved significantly more weight loss during follow-up compared to their matched controls (*p* < 0.001).

The baseline demographic, clinical, and treatment characteristics of patients by cohort are described in Table 1.

**Table 1:** Baseline demographic, clinical, and treatment characteristics of the patients divided by surgery status

Characteristics	No bariatric surgery (*n* = 2959)	Bariatric surgery (*n* = 2959)	*p*=
Demographic			
Age (mean, SD)	44.8 (13.1) years	44.6 (10.6) years	0.311
BMI (mean, SD)	41.2 (9.1) kg/m^2^	41.2 (7.2) kg/m^2^	0.371
Male	506 (17.1%)	645 (21.8%)	< 0.001
Δ Weight during follow-up (mean, SD)	1.4 (14.0) kg	− 24.5 (25.3) kg	< 0.001
Clinical			
Hypertension (*n*, %)	773 (26.1%)	861 (29.1%)	0.011
Resolved (*n*,%)	67/773 (8.7%)	80/861 (9.3%)	0.316
Hyperlipidaemia (*n*, %)	161 (5.4%)	194 (6.6%)	0.080
Resolved (*n*,%)	27/161 (16.8%)	49194 (25.3%)	0.015
Diabetes (*n*, %)	654 (22.1%)	750 (25.4%)	0.004
Resolved (*n*,%)	34/654 (5.2%)	39/750 (5.2%)	0.638
Atrial Fibrillation (*n*, %)	1 (0.03%)	25 (0.8%)	< 0.001
Smoking (*n*, %)	1079 (36.5%)	1076 (36.4%)	0.957
Alcohol (*n*, %)	422 (14.3%)	539 (18.2%)	< 0.001
Treatment			
Warfarin (*n*, %)	176 (6.0%)	125 (4.2%)	0.003
Direct oral anticoagulant (*n*, %)	26 (0.88%)	25 (0.84%)	1.000
Aspirin (*n*, %)	166 (5.6%)	783 (26.5%)	< 0.001
Statin (*n*, %)	166 (5.6%)	783 (26.5%)	< 0.001
ACE inhibitors *(n*,%)	44 (1.5%)	280 (9.5%)	< 0.001
B-Blockers (n,%)	187 (6.3%)	482 (16.3%)	< 0.001
Oral Hypoglycaemic (n,%)	221 (7.5%)	729 (24.6%)	0.000
Follow-up			
Follow-up (mean, SD)	130.3 (85.6) months	52.7 (39.8) months	< 0.001

### Primary endpoint

**Fig. 2:**
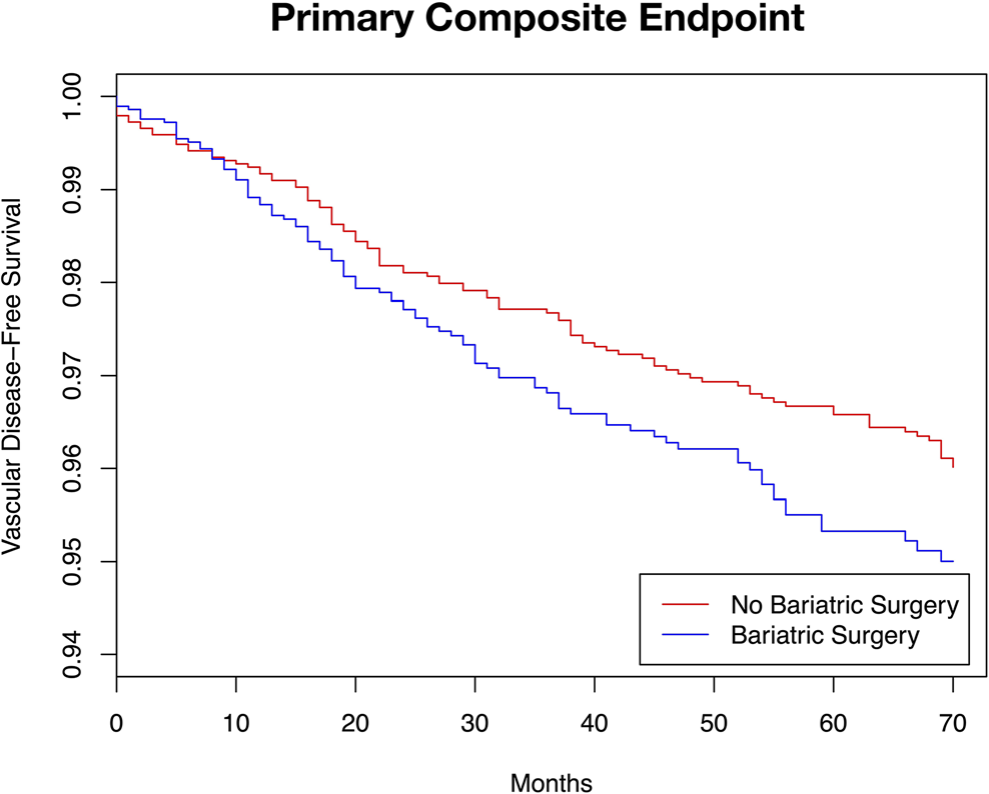
Adjusted primary endpoint rates by bariatric surgery status during follow-up time

The primary endpoint of all types of peripheral vascular disease (arterial disease, venous disease) occurred in a total of 346 patients. Patients in the bariatric surgery group had similar event rates (HR = 1.223, 95%CI 0.947–1.580, *p* = 0.124). The results of the analysis are outlined in Table 2 and depicted in Fig. 2.

**Table 2:** Endpoint occurrence during follow-up

Events (per 10,000 person-years)	No bariatric surgery (*n* = 2959	Bariatric surgery (*n* = 2959)	HR	95% CI	*p* **=**
Primary outcomes					
Primary outcome: peripheral vascular disease^a^ (/10,000 py)	6.0	7.5	1.223	0.947–1.580	0.124
Peripheral arterial disease^b^ (/10,000 py)	2.6	1.3	0.560	0.327–0.959	0.035
Peripheral venous disease^c^ (/10,000 py)	3.7	6.3	1.685	1.256–2.262	< 0.001

^a^Peripheral vascular disease events: composite of arterial disease, and venous disease

^b^All peripheral arterial disease outcomes: composite of claudication, angina cruris, arterial insufficiency, limb ischaemic (acute or chronic), arterial ulcer, ABPI < 0.8

^c^All peripheral venous disease outcomes: composite of venous ulcer, venous insufficiency, varicose veins, venous eczema

### Secondary endpoint

**Fig. 3:**
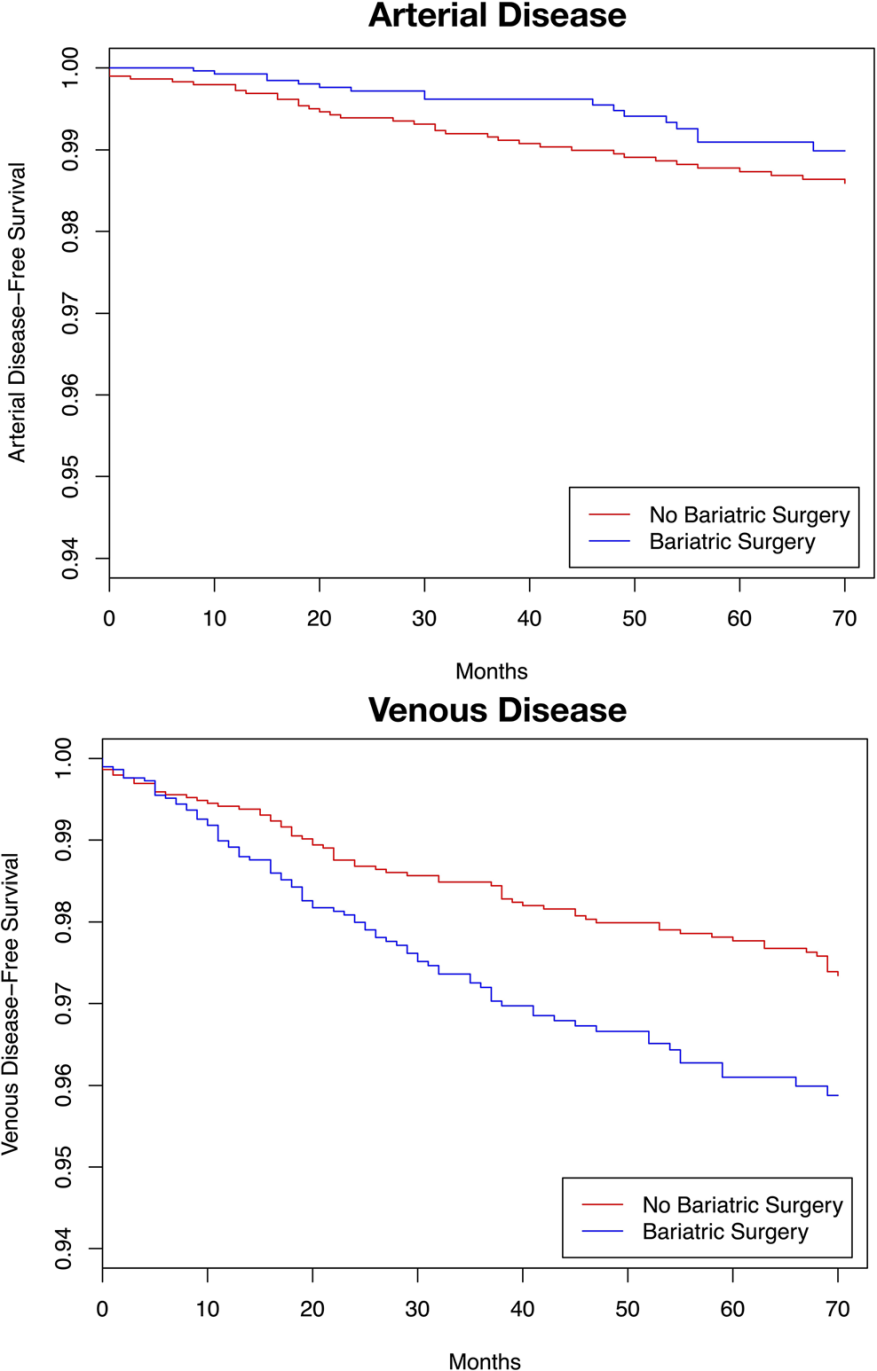
Adjusted secondary endpoint rates by bariatric surgery status during follow-up time

A total of 122 patients had a diagnosis of peripheral arterial disease during follow-up; a total of 243 patients developed lower limb venous disease (Fig. 3).

Peripheral arterial disease was experienced by 21 of the bariatric surgery patients and 101 of the controls, with rates of 1.3 and 2.6 events per 10,000 patient years respectively (HR = 0.560, 95%CI 0.327–0.959, *p* = 0.035). Ninety-nine in the bariatric surgery cohort and 144 of the controls received a diagnosis of lower limb venous disease during follow-up, translating to a rate of 6.3 and 3.7 events per 10,000 patient years respectively (HR = 1.685, 95%CI 1.256–2.262, *p* < 0.001). The results of the analyses are displayed in Table 2.

### Sensitivity and subgroup analyses

Two sensitivity analyses were carried out. Firstly, without adjustment for covariates and secondly only including patients who had gastric bypass and gastric banding. Both analyses yielded similar results to primary analysis (Table 3).

**Table 3:** Sensitivity analysis for sleeve gastrectomy and gastric bypass surgery

	HR	95% CI	*p* =
Sensitivity model 1: unadjusted model			
Primary outcome: peripheral vascular disease^a^	1.190	0.932–1.520	0.164
Peripheral arterial disease^b^	0.584	0.354–0.962	0.035
Peripheral venous disease^c^	1.603	1.207–2.129	0.001
Sensitivity model 2: including sleeve gastrectomy and gastric bypass only			
Primary outcome: peripheral vascular disease^a^	1.026	0.734–1.434	0.881
Peripheral arterial disease^b^	0.336	0.133–0.847	0.021
Peripheral venous disease^c^	1.380	0.961–1.981	0.081

^a^Peripheral vascular disease events: composite of arterial disease, and venous disease

^b^All peripheral arterial disease outcomes: composite of claudication, angina cruris, arterial insufficiency, limb ischaemic (acute or chronic), arterial ulcer, ABPI < 0.8

^c^All peripheral venous disease outcomes: composite of venous ulcer, venous insufficiency, varicose veins, venous eczema

Interaction analysis revealed a stronger effect size in patients of higher BMI classes at index (*p* = 0.229); but no significant interaction with gender was present (*p* = 0.384) as displayed in Table 3. There was no difference in rates of primary events across different bariatric surgery types (*p* = 0.767), as displayed in Table 4.

**Table 4:** Primary outcome occurrence by bariatric surgery type

Bariatric surgery type	Number of patients (*n* = 2959) (*n*)	Number of patients with primary events (*n*,%))	*p* = (logrank)
Gastric banding	1147	51 (4.4%)	0.767
Gastric bypass	1207	39 (3.23%)	
Sleeve gastrectomy	432	6 (1.38%)	
Duodenal switch	21	0	
Gastric stapling	7	2 (28.5%)	
Stomach partitioning	5	0	
Mason vertical banded gastroplasty	4	0	
Undefined bariatric surgery	136	17 (12.5%)	

## Discussion

Bariatric surgery is a growing field that is rapidly becoming a major cornerstone in the management of patients with obesity. Its use in the UK has grown precipitously over the last decade: in a recent epidemiological study based on CPRD data, the number of surgeries performed in the 2010–2012 interval was found to be more than nine times higher than in the 2002–2005 interval. However, despite its growing use, much remain unanswered with regards to its long-term clinical impact. Does it truly reduce obesity-related morbidity in the long-term or does it only provide temporary benefit? Do the potential long-term benefits outweigh the short-term risks of the surgical procedure and cost?

Because of its relatively short history, evidence on the long-term effects of the surgery is currently still scarce, with only a handful of promising studies achieving adequate cohort sizes and long follow-up time. This study presents data that adds to this growing pool of evidence with one of the largest bariatric cohorts reported on to date, addressing the lower limb arterial and venous outcomes of 2,959 patients in the CPRD. The results of the study reveal similar overall rates of peripheral vascular morbidity but reveal a lower rate of arterial disease among the bariatric cohort which is offset by higher rates of venous disease. Considering the high vascular morbidity that is associated with obesity, and the relative gravity of arterial disease when compared to venous disease, the potential beneficial effect of bariatric surgery on arterial outcomes reported in this study may translate to an important clinical benefit in the current obesity epidemic.

There are a number of possible interpretations for the study results. Firstly, the reduced rate of peripheral arterial disease detected in the bariatric surgery patients is likely to relate to an improvement in risk factor control. As evidenced by the higher rates of resolution of risk factors in the bariatric surgery cohort, and supported by prior literature on the subject [3, 16], this is the most likely underlying explanation. This result is in concordance with previous literature that has detected lower rates of atherosclerotic disease in a number of bariatric surgery cohorts, in the context of coronary, and other macrovascular diseases [17, 18]. A further potential contributing mechanism for this is that bariatric surgery is known to be associated with lower rates of incident AF and higher rates of AF resolution [19, 20], which may contribute to lower arterial thrombosis rates.

On the other hand, the rates of venous disease were higher among the bariatric surgery cohort. This goes against the hypothesis we provided for our study, whereby lower intra-abdominal pressure was thought to have the potential to contribute to better venous return and therefore lower rates of peripheral venous disease. There are a number of potential explanations for this discrepancy. Firstly, and most importantly, it is likely that this endpoint is subject to a degree of measurement bias. As patients who undergo bariatric surgery are subject to stricter and more frequent follow-up, the increase in medical contact provides higher opportunity to pick up symptoms from venous disease. Secondly, it is possible that the reduction in mobility perioperatively, and concordant risk of venous thrombosis also plays a role in the increased rates of venous disease. However, we would expect this impact to be minor in view of the long follow-up time in this study.

### Limitations

Due to data coding reasons, it was not possible for us to discern amputations secondary to lower limb vascular disease, as opposed to those due to traumatic, infectious, malignant or other causes. For this reason, amputation was not considered as an endpoint in this study. Similarly, we are unable to determine the exact symptomology that clinically coded diagnoses correspond to, or extract information regarding specific interventions performed for PAD. However, considering the primary endpoint is the occurrence of new PAD, this rate should be shielded from impact. Further evaluation, in the context of a database specifically designed to investigate and distinguish the rates of lower limb vascular disease-related amputations may be warranted. Furthermore, due to lack of data granularity, it was not possible to accurately establish dosing and timing of medication use among patients. This is an important limitation, and further assessment in a database that contains suitable detail for this purpose is warranted.

The CPRD has been previously widely validated as being accurately representative of the UK population for demographic characteristics such as age, sex, morbidities, and ethnicity. This implies that the results of this study are likely to be generalisable to any Western country that has similar demographic characteristics to the UK. A benefit of this study is the large number of patients included, which improves the reliability of the data and the conclusions that are drawn from it. However, its design also presents some limitations. The medical records used for the database are totally anonymised, and it is therefore not feasible to fill or research missing follow-up data. The follow-up is therefore strictly limited to permanence in the database which is in part geographically determined. Furthermore, as the data collection is linked to primary- and secondary-care diagnostic data, its accuracy is entirely reliant on the accuracy of the clinicians’ diagnosis, and on the accuracy of data coding and transcription into the database. Additionally, the CPRD population with obesity is predominantly female; this has implications for the external validity of the study allowing the results to be generalisable more to females than to males. In order to reduce the potential bias that this may have, we have matched the cohorts on variables including sex.

## Conclusion

In conclusion, this study provides evidence on the long-term lower limb vascular morbidity of patients with obesity who undergo bariatric surgery. While we did not observe a significant protective effect of bariatric surgery on peripheral vascular disease as a whole, we did find a significant reduction in arterial morbidity in patients who underwent bariatric surgery when compared to propensity-score-matched controls that did not. In contrast, we also noted a significant increase in venous disease in bariatric surgery patients, which may relate to an increased rate of detection due to the higher degree of contact with routine care in patients who undergo the surgery. The findings of the study are important both for immediate clinical practice and to guide further research. With the wealth of emerging evidence regarding the benefits of bariatric surgery, that range from mental health to the resolution of diabetes, the indications for bariatric surgery, and clinician awareness of its benefits is growing, but evidence still has a long way to go. These results highlight the requirement for a large-scale, randomised controlled trial investigating the long-term effect of bariatric surgery, in order to validate the emerging results from retrospective studies. Furthermore, the issue of takeup must be considered: less than 1% of patients who are eligible for bariatric surgery currently undergo it. Considering the potential benefits displayed in this study, combined with the wealth of evidence that suggests reductions in cancer-related mortality, diabetes, all-cause mortality and many more, the lack of access to care experienced by the 99% of eligible patients may represent avoidable, long-term adverse clinical outcomes. Overall, it is time to consider the factors that are contributing to this unmet clinical need, in the interest of improving the long-term outcomes of patients with obesity.

## Supplementary Information

Supplementary Figure 1 Propensity density plots (PNG 330 kb).
