# Supplementary figures and images for: Long-term incidence and outcomes of obesity-related peripheral vascular disease after bariatric surgery
Source: Langenbecks Arch Surg. 2021 Jan 12;406(4):1029–36. doi: 10.1007/s00423-020-02066-9 (PMC8208905; doi:10.1007/s00423-020-02066-9)

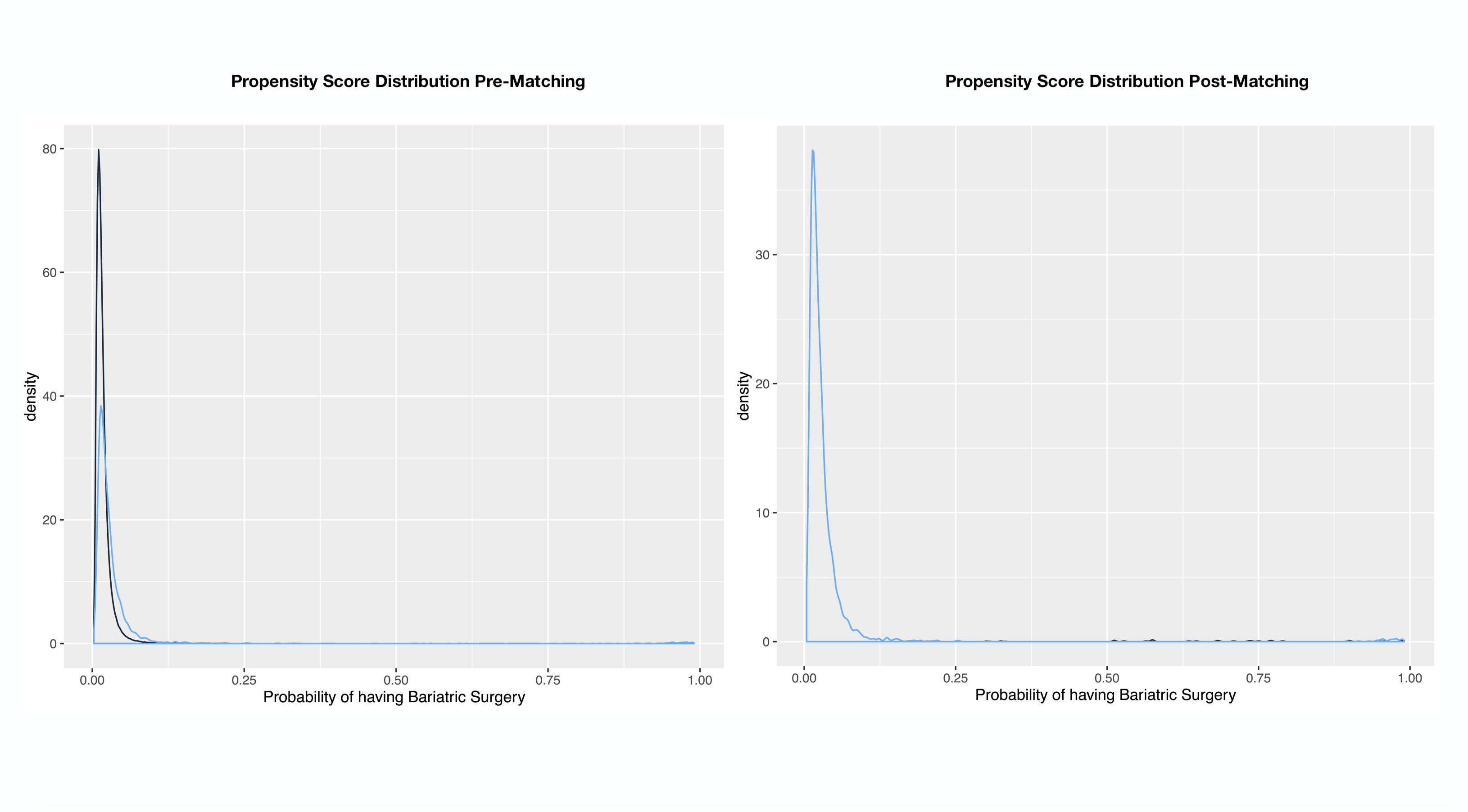

Supplement: Supplementary file 1 — Propensity density plots (PNG 330 kb). [file 423_2020_2066_MOESM1_ESM.png]
